# Case report: Primary vulvar adenocarcinoma of mammary gland type—its genetic characteristics by focused next-generation sequencing

**DOI:** 10.3389/pore.2024.1611376

**Published:** 2024-03-20

**Authors:** Lina Hu, James Tiesinga

**Affiliations:** Alaska Native Medical Center, Department of Pathology, Anchorage, AK, United States

**Keywords:** MLVA, mutations by NGS, immune markers, triple positive, immunohistochemical features

## Abstract

Mammary-like vulvar adenocarcinoma (MLVA) is an exceedingly rare subtype of vulvar adenocarcinoma that shares features with mammary gland tissue. Due to its rarity and lack of consensus, MLVA presents diagnostic challenges to pathologists. We present the case of a 59-year-old female with an ulcerated mass on the right side of the external genitalia, diagnosed as MLVA. Comprehensive immunohistochemistry (IHC) and gene sequencing studies were performed to characterize the tumor. IHC analysis revealed triple expression of hormonal receptors (estrogen receptor, progesterone receptor, and HER2), supporting the mammary gland origin of the tumor. Gene sequencing identified unique genetic mutations associated with the expression of hormonal markers. One fusion gene (ERBB2-NAGLU) has not been reported in any tumors, and other mutations with unique mutation types have not been previously reported in MLVA. Our findings shed light on the molecular characteristics of MLV and may help improve the diagnosis and treatment of this rare type of vulvar adenocarcinoma.

## Introduction

Primary vulvar adenocarcinomas are a rare heterogeneous group of tumors that can be classified based on organ origin (Bartholin gland, skene gland, mammary gland, or sweat gland) and histomorphology (clear cell carcinoma, adenocarcinoma of cloacogenic type, apocrine adenocarcinoma, adenoid cystic carcinoma or extramammary Paget’s disease with invasive adenocarcinoma, etc.). Among vulvar adenocarcinomas, mammary gland type (MLVA) is exceedingly rare, and often presents diagnostic challenges. To better understand the basic features of this rare type of adenocarcinoma, we conducted comprehensive immunohistochemistry and gene sequencing studies. Our analysis revealed that the case exhibited IHC features of mammary gland with triple expression of hormonal receptors and unique genetic mutations related to these hormonal marker expressions. This is the second triple positive MLVA case detected and the second case of MLVA with gene sequencing information. Most of the genomic mutations found in our case are similar to that of luminal B breast cancer and the mutation types are different from the only MLVA case with sequencing data [1] even though the same genes are involved.

## Case description

59-year-old female, Gravida 6, para 5 with external genitalia showing an ulceration ∼5 cm on the right side at the low posterior level of the urethra. This mass is ∼7 cm wide and 12 cm anterior to posterior extending along the vulva on the right side up to the level of the clitoris. On speculum exam, the cervix is normal in appearance. There is no area of leukoplakia or evidence of dysplasia beyond the open ulcer. The area of the ulcerated wound was biopsied by taking the firm part of the lesion, 1 cm cube in all directions. The CT of the abdomen and pelvis shows multiple enlarged right inguinal lymph nodes, the largest one measuring 5.0 cm × 4.6 cm × 3.2 cm. There are also multiple right external iliac chain lymph nodes, the largest measuring 2.9 cm × 1.9 cm, consistent with lymph node metastasis. There is no history of primary breast cancer or uterine malignancy. The only surgical history is bilateral tubal ligation.

**FIGURE 1:**
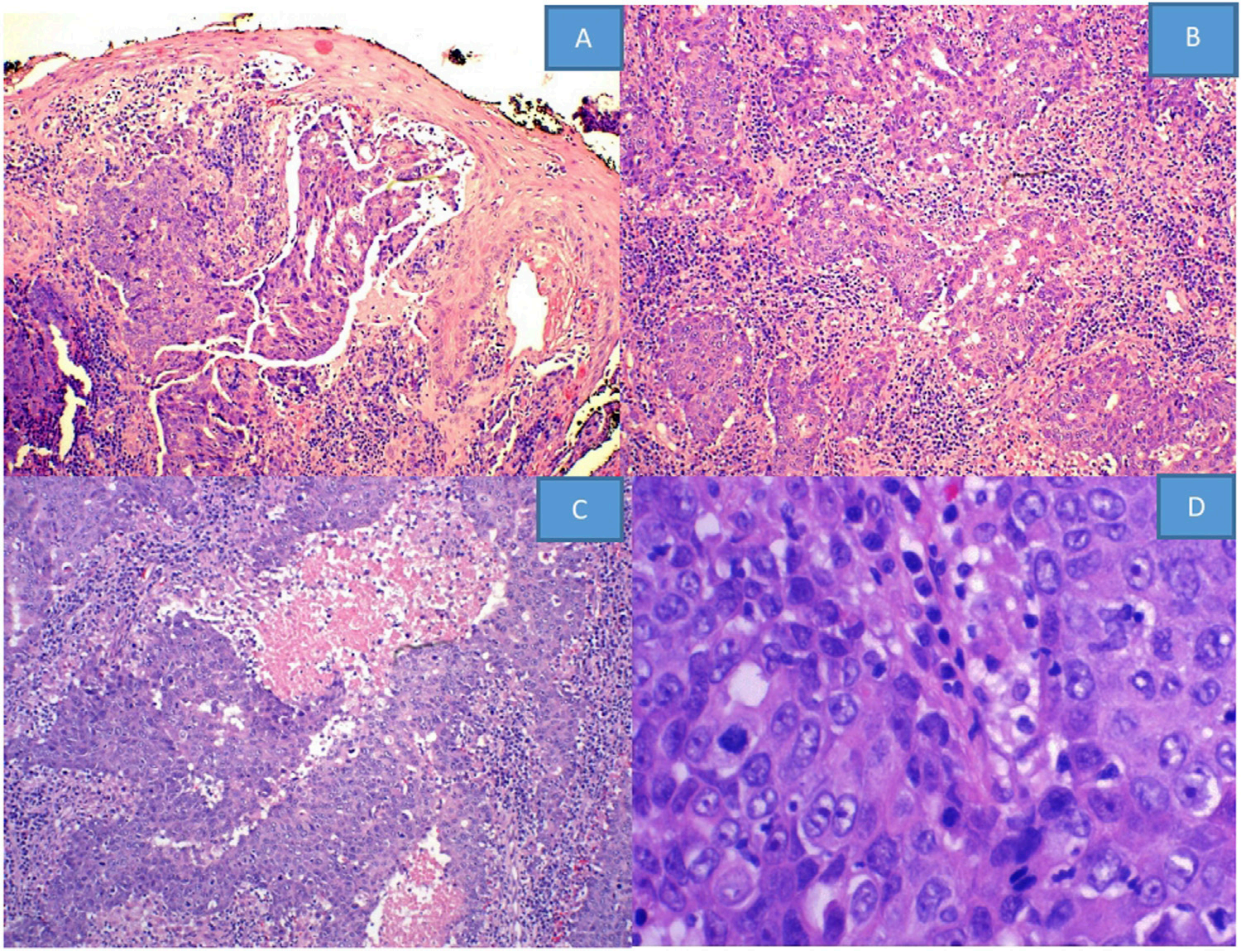
HE stained slides from **(A–D)** show: **(A)** squamous epithelium with no Pagetoid changes, while the dermal tumor exhibits a vaguely glandular structure (×10). **(B)** Shows infiltrative tumor cells forming irregular nests with stromal inflammatory reaction and **(C)** with geographic necrosis (×20). **(D)** Demonstrates vesicular nuclei with aberrant mitotic figures (×40).

Pathological review of HE stained slides shows nested vaguely formed glands with patchy necrosis, lymphocytic infiltration and frequent mitoses (Figure 1).

IHC staining for CK7, GATA3, ER, Ki67, CK20, CK5/6, P40, p63, PR, GCDFP-15, STAB2, villin, mammaglobin, PAX8, p40, HER2, and FISH HER2 are performed.

Next-generation sequencing was conducted using the OmniSeq Insight technique aimed at all coding exons from 523 cancer related genes and select regions from 55 commonly rearranged genes.

**FIGURE 2:**
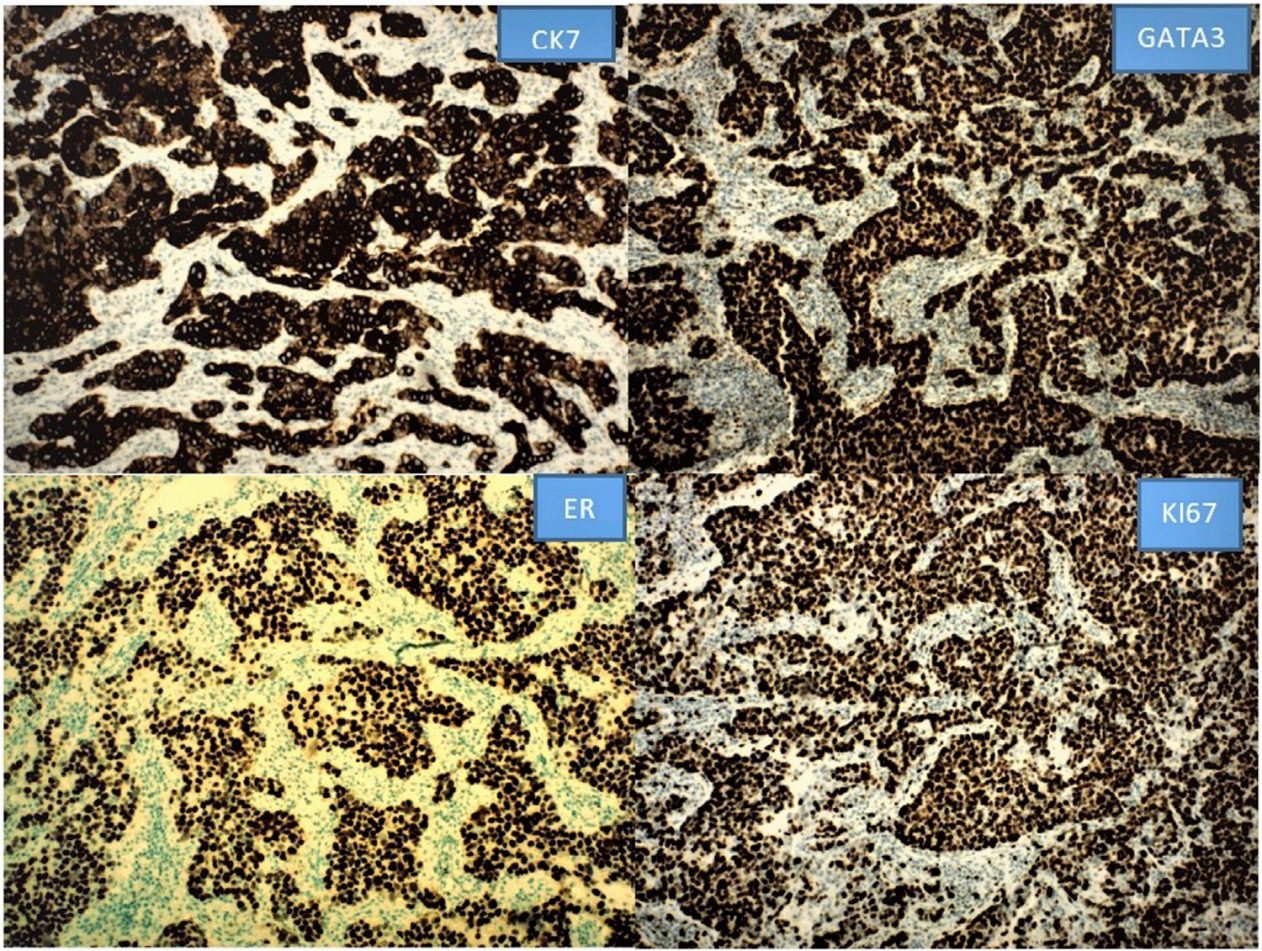
The positive IHC staining for CK7, GATA3 and ER with high Ki67 labeling index is illustrated as below. Other stains are not shown.

IHC staining for CK7, GATA3, and ER shows strong positivity for CK7, GATA3, and ER with proliferation index by Ki67 over 90% (Figure 2). There is variably positivity for mammaglobin, CK5/6 and p40; 5% tumor cells positive for PR, and equivocal for HER2; but FISH HER2 is amplified in a ratio of 8.5 (positive); negative for GCDFP-15, CK20, SATB2, villin, PAX8, and P63. Negative staining for CK20, SATB2, villin and PAX8 argues against colorectal, urothelial and Mullerian primary; negative P63 and focal positivity on CK5/6 and p40 argues against squamous cell carcinoma. The findings of positive CK7, GATA3, ER, PR, mammaglobin, and HER2 FISH amplification support the diagnosis of MLVA.

The five genomic variants detected are ERBB2-NAGLU fusion at ERBB2 exon 15 and intron 1 of NAGLU, (HER 2- N-acetyl-alpha-glucosaminidase), ESR1-CCDC170 fusion, (estrogen receptor gene and coiled-coil domain containing 170), MYC gain, PIK3CA p104_G106delinsR and TP53 c1180_*1delTGAC.

## Discussion

Vulvar adenocarcinomas comprising less than 0.1% of vulvar malignancies [2], and adenocarcinoma derived from mammary-like glands is even rarer. Since the first reported case in 1936 until 2022, only 41 cases of MLVA have been described [3–6]. Our immunohistochemical studies indicate that MLVA arises from mammary-type glands in the vulva. The mammary-like glands in the vulva consist of coiled tubules giving rise to glandular acini lined by simple columnar epithelium with apical snouts. These glands differ from normal sweat glands by showing positivity for estrogen and progesterone receptors, and they are distinct from both sweat and mammary glands based on ultrastructural studies. Tumors arising from these glands exhibit certain features resembling mammary glands and can be classified into four intrinsic subtypes based on receptor expression: luminal A, luminal B, HER2-enriched, and basal-like. The histomorphological features of mammary-like vulvar adenocarcinoma range from well-differentiated to poorly differentiated adenocarcinoma. Poorly differentiated MLVA shows large pleomorphic epithelioid cells forming nests and cords with hyperchromatic nuclei, lacking definite gland formation, papillary structures, or intraluminal vacuoles. Well-differentiated tumors exhibit a cribriform or tubular architecture with small, infiltrative glands scattered in a fibrotic stroma and a low mitotic count. To diagnose mammary-like vulvar adenocarcinoma, proposed criteria [5] include morphological similarity to breast carcinoma, positive estrogen receptor and/or progesterone receptor status, positivity for typical immunohistochemical breast markers, and the presence of carcinoma *in situ* or non-neoplastic breast tissue adjacent to the tumor, after excluding primary breast carcinoma or metastasis from other organs. However, these criteria may only be applicable to well-differentiated MLVA, as our case does not exhibit morphological similarity to breast cancer, nor the presence of carcinoma *in situ* or non-neoplastic breast tissue adjacent to the tumor. Therefore, immunohistochemical staining may play a crucial role in establishing the diagnosis. However, there is no single specific marker for MLVA. A panel is necessary for accurate diagnosis of the entity. Case reports have documented the application of various markers including CK7, CK20, ER, PR, HER2, BRST1, BRST2 (GCDP15), CEA, BCL2, CAM5.2 P53, Mib1, S100, HMB45, AE1/3, SOX10, E-Cadherin, P63, GATA3, TTF1, calponin, vimentin, CDX2, CK5/6, PAX8, and P16. It is evident that many of these markers were used to resolve the differential diagnosis. In our study, we employed additional markers such as STAB2, villin, mammaglobin, and P40. Based on our results and the available literature, we can confidently classify MLVA as a CK7-positive, CK20-negative tumor with variable expression of hormonal receptors. The positive GATA3 and mammaglobin staining further support its mammary gland like features. In fact, the expression of hormonal receptors in MLVA closely resembles that of breast cancers, which can be ER+/PR−/HER2−, ER+/PR+/HER2−, ER−/PR−/HER2+, triple-negative, or, as in our case, triple-positive—the second MLVA case detected as such. Because of strong ER expression, weak PR and HER2 amplification, our case resembles the breast carcinoma of luminal B type.

As for genomic alterations, only one article has provided a detailed description of the molecular and genetic alterations observed in MLVA [1]. The genetic mutations identified through our targeted gene sequencing align with the current understanding of the molecular characteristics of breast cancer. Notably, well-known genetic alterations implicated in breast cancer development and progression, such as ESR1-CCDC170 fusion, PIK3CA mutations, TP53 mutations, and MYC gain, were also identified in our study.

Our studies indicate that the ERBB2-NAGLU fusion occurs at exon 15 of the ERBB2 gene and intron 1 of the NAGLU gene. Exons 13–16 encompass the growth factor receptor domain IV, with exon 15 being part of this domain. Exon 15 encodes part of the intracellular kinase domain of HER2. The kinase domain is critical for the catalytic activity of HER2 and is involved in transmitting signals downstream by phosphorylating specific target proteins. The kinase activity of HER2 participates in signaling cascades that regulate cell growth, survival, and differentiation. Mutations within these exons can affect the structure and function of HER2, potentially leading to dysregulated signaling and contributing to cancer development or progression. The ERBB2-NAGLU fusion has not been reported before in any tumors and its significance is not yet fully understood although separately each gene has been well studied. ERBB2, also known as HER2, is a gene encoding a cell surface receptor protein involved in cell growth and division. On the other hand, NAGLU encodes an enzyme responsible for degrading heparan sulfate, a large sugar molecule in the body, by hydrolyzing terminal N-acetyl-D-glucosamine residues. The fusion of these genes could theoretically result in a hybrid gene by combining their coding regions, potentially leading to the production of a novel protein with altered biological function. However, the role of the ERBB2-NAGLU fusion involving ERBB1 exon 15 and NAGLU intron 1 remains unclear, as the intron itself does not interrupt the gene sequences. However, noncoding introns can be spliced out during mRNA processing, which could potentially result in a chimeric protein containing segments from both ERBB and NAGLU proteins, leading to altered protein function.

The ESR1-CCDC170 fusion is considered a gain-of-function mutation that enhances the ligand-independent growth factor signaling pathway, leading to increased cell aggressiveness and tumorigenesis [7]. It is the most frequently detected gene fusion in luminal B breast cancer. ESR1-CCDC170 binds to HER2/HER3/SRC and activates the SRC/PI3K/AKT downstream signaling pathway, promoting cancer cell survival and contributing to tumor aggressiveness. The presence of ESR1-CCDC170 fusion variants can reduce endocrine sensitivity and confer resistance to tamoxifen [8]. Moreover, the fusion between ESR1 and CCDC170 has been shown to predict shorter disease-free survival and overall survival [9].

PIK3CA (phosphatidylinositol-4,5-bisphosphate 3-kinase, catalytic subunit alpha) is a gene that encodes the catalytic subunit of PI3K (phosphoinositide 3-linase, also known as the p110α). The PIK3CA gene provides instructions for producing the p110α proteins. The P110α is involved in the activation of PI3K signaling pathway. PIK3CA mutations are among the most common genetic aberrations in breast cancer, occurring in over one-third of cases [10]. Phosphatidyl 3-kinases (PI3K) are a family of lipid kinases involving cell growth, proliferation, differentiation, motility, and survival. They are particularly enriched in luminal and human epidermal growth factor receptor 2-positive subtypes. The specific mutation PIK3CA p104_G106delinsR found in our case refers to a mutation in the PIK3CA gene, which encodes the p110α subunit of PI3K protein. This mutation involves the deletion of amino acids 104 to 106 (glycine-serine-histidine) and the insertion of arginine at the same location and is classified as an activating mutation, as it enhances the activity of the PI3K signaling pathway and elevated PI3K signaling is considered a hallmark of cancer. P104_G106delinsR has been reported in certain cases of breast cancer.

The TP53 c1180_*1delTGAC mutation refers to the deletion of the nucleotides TGAC at position 1180, resulting in a frameshift mutation specifically in the intronic region downstream of exon 10. As a result, the deletion affects the sequence downstream of position 1180. It may disrupt splicing, mRNA stability, or other regulatory mechanisms. The c1180_*1delTGAC mutation has been observed in some cases of breast cancer and is considered a loss-of-function mutation, leading to a decrease or loss of p53 protein activity. The p53 protein plays a vital role in numerous cellular processes, including DNA repair, cell cycle arrest, and apoptosis. The loss of p53 function can result in genomic instability and an increased risk of cancer development and progression. Both TP53 and PIK3CA are among the most frequent mutated genes in luminal B breast cancers [11].

Another mutation present in our MLVA case is MYC gain, which involves the amplification or overexpression of the MYC gene. MYC is a proto-oncogene that encodes a nuclear phosphoprotein involved in cell proliferation, differentiation, apoptosis, and cellular transformation. MYC gain results in an elevated level of MYC protein in cancer cells. This genetic alteration is well-known in adenocarcinoma and has been associated with aggressive tumor behavior and unfavorable clinical outcomes. MYC amplification is most commonly observed in the basal-like subtype of breast cancer. However, MYC overexpression has also been identified in other subtypes, including HER2-positive and luminal B subtypes.

In luminal B breast cancer, MYC overexpression is linked to a poorer prognosis and an increased risk of recurrence. In summary, MYC gain, whether through amplification or overexpression, is associated with an adverse prognosis and aggressive behavior in breast cancer. Its significance may be similar in the context of MLVA.

Comparing our findings with published sequencing data [1], three out of the six key genes of interest—ERBB2, PIK3CA, and TP53 mutations—are also present in our case, albeit with differences in the types of mutations. For TP53, we identified a frameshift mutation instead of a missense mutation. Regarding ERBB2, we detected a structural rearrangement rather than a point mutation. As for PIK3CA, we found an in-frame deletion and insertion mutation instead of a point mutation. Therefore, our molecular findings in MLVA are somewhat unique. Coincidentally, this published sequencing data was also performed in a poorly differentiated mammary like vulvar adenocarcinoma.

## Conclusion

Multiple gene mutations are involved in tumorigenesis and development of MLVA. Our MLVA case has demonstrated a resemblance to HER2+ luminal B breast cancer by its hormonal marker expressions and genetic mutations. In consideration of the overexpression, gain-of-function mutation, and estimated copy number gain, it is possible that ERBB2 could be a driver mutation in this case. Many of these mutations have been the focus of targeted therapies, including an existing agent for HER2, and development for further investigation is required to fully understand its role and implications in MLVA.

## Data Availability

The original contributions presented in the study are included in the article/supplementary material, further inquiries can be directed to the corresponding author.
